# Temporal Processing of Joyful and Disgusting Food Pictures by Women With an Eating Disorder

**DOI:** 10.3389/fnhum.2018.00129

**Published:** 2018-04-06

**Authors:** Caroline Gagnon, Catherine Bégin, Vincent Laflamme, Simon Grondin

**Affiliations:** École de Psychologie, Université Laval, Québec, QC, Canada

**Keywords:** time perception, emotion, eating disorders, food, bisection, discrimination

## Abstract

The present study used the presentation of food pictures and judgements about their duration to assess the emotions elicited by food in women suffering from an eating disorder (ED). Twenty-three women diagnosed with an ED, namely anorexia (AN) or bulimia nervosa (BN), and 23 healthy controls (HC) completed a temporal bisection task and a duration discrimination task. Intervals were marked with emotionally pre-rated pictures of joyful and disgusting food, and pictures of neutral objects. The results showed that, in the bisection task, AN women overestimated the duration of food pictures in comparison to neutral ones. Also, compared to participants with BN, they perceived the duration of joyful food pictures as longer, and tended to overestimate the duration of the disgusting ones. These effects on perceived duration suggest that AN women experienced an intense reaction of fear when they were confronted to food pictures. More precisely, by having elevated the arousal level and activated the defensive system, food pictures seemed to have speeded up the rhythm of the AN participants’ internal clock, which led to an overestimation of images’ duration. In addition, the results revealed that, in both tasks, ED women presented a lower temporal sensitivity than HC, which was related to their ED symptomatology (i.e., BMI, restraint and concern) and, particularly, to their weaker cognitive abilities in terms of attention, processing speed and working memory. Considered all together, the findings of the present experiment highlight the role of fear and anxiety in the manifestations of AN and point out the importance of considering non-temporal factors in the interpretation of time perception performance.

## Introduction

Time perception refers to the subjective experience of time, which indicates how a person interprets the duration of an event. The sense of time is modulated by intrinsic (e.g., age, gender, menstrual cycle; Block et al., 2000; Morita et al., 2005; Glicksohn and Hadad, 2012; Pütz et al., 2012; Ferreira et al., 2016) and extrinsic contexts (e.g., medication, drugs, rhythmical stimuli; Droit-Volet et al., 2010; Lake and Meck, 2013; Shahabifar and Movahedinia, 2016). Among the intrinsic factors, emotions are particularly important. As suggested by the saying “time flies when we are having fun,” time perception is intimately connected to emotional life (Droit-Volet and Gil, 2009).

Viewed from a pacemaker-accumulator (internal-clock) interpretation of time processing (Gibbon, 1977; Gibbon et al., 1984), emotions can affect time perception through two of the latter’s components: the pacemaker or the attentional mechanisms. Several researches have shown that an increase of the arousal level generates a relative lengthening effect of perceived duration (e.g., Wearden and Penton-Voak, 1995; Droit-Volet and Wearden, 2002; MacDonald and Meck, 2005). More precisely, increased arousal speeds up the rhythm of the pulses’ emission of the pacemaker. If more pulses are amassed in the accumulator, then time seems longer. Therefore, by producing an elevation of arousal, an emotional event should make the internal clock run faster, leading to an overestimation of stimulus duration (Gil and Droit-Volet, 2012). For instance, Droit-Volet et al. (2004) showed that the presentation durations of emotional faces depicting anger, sadness or happiness were perceived longer than the ones of neutral faces. In parallel, the number of pulses collected would be under the control of attention, with more attention allowing a larger accumulation (Block and Zakay, 1996; Zakay and Block, 1996, 1997). Thus, assuming that there is a limited pool of attentional resources, being distracted from the passage of time leads to a lower number of pulses reaching the accumulator and to a shortening effect (i.e., durations are underestimated; for review see Lejeune, 1998; Brown, 2008). For example, Gil and Droit-Volet (2011a) showed that the presentation durations of ashamed faces were underestimated in comparison to those of neutral ones (see also Grondin et al., 2015). The feeling of shame could direct the focus of attention on a person’s thoughts about the self or on causes of shame (i.e., self-awareness, reflexive activity; Lewis, 1971), and thus deviate the attentional resources from the events to be timed.

Interestingly, Angrilli et al. (1997) proposed an integrative view of the influence of emotions on time perception. They reported that perceived duration depends on an interaction between arousal and attentional processes. Precisely, on low-arousing conditions, the duration of negative stimuli is underestimated, whereas the duration of positive stimuli is overestimated. On high-arousing conditions, opposite results are found: the duration of negative stimuli is overestimated, whereas the duration of positive stimuli is underestimated. In consequence, according to Angrilli et al. (1997), two causal forces for the effect of emotions on perceived duration seem to coexist: a controlled-attentional mechanism for low-arousal conditions (i.e., negative pictures distract more the attentional resources from the passage of time than positive ones), and an automatic emotion-driven mechanism for high-arousing conditions (i.e., negative stimuli accelerate more the rhythm of the pacemaker than positive ones). Based on the work of LeDoux (1995) on fear processing, the authors explained that negative and high-arousing stimuli, by speeding up the rhythm of the internal clock, activate the defensive system by quickly and automatically processing dangerous cues and preparing the responses programs associated to fear behaviors. In other words, the acceleration of the pacemaker in the presence of an intense stress or a threatening situation could have a motivational-survival function (Bradley et al., 2001).

Over the years, a variety of stimuli have been used to elicit emotions in time perception studies: facial expressions (Effron et al., 2006; Gil et al., 2007; Gan et al., 2009; Tipples et al., 2015; Mioni et al., 2016a), music pieces (Bisson et al., 2009; Droit-Volet et al., 2010, 2013), video sequences (Droit-Volet et al., 2011; Grondin et al., 2014a), sounds (Noulhiane et al., 2007; Mioni et al., 2017), words (Zhang et al., 2017), images of mutilated bodies (Grondin et al., 2014b), phobic objects (Watts and Sharrock, 1984; Buetti and Lleras, 2012; Tipples, 2015) and real-life scenes (Lambrechts et al., 2011). Gil et al. (2009) first used food pictures to test the effect of disgust and pleasure on time perception. With a temporal bisection task, they found that the duration of disgusting and pleasant food pictures was underestimated by healthy participants in comparison with a neutral stimulus, and that this shortening effect was more marked for the disgusting pictures than for the liked ones. They attributed their results to an attentional distraction from the passage of time caused by food images, especially the disliked ones.

Food represents a type of stimuli particularly sensitive for people suffering from an eating disorder (ED) like anorexia (AN) and bulimia nervosa (BN). In fact, when presented with food, women with an ED experience less pleasure and strong negative emotions like fear, disgust and lack of control (Gorini et al., 2010; Giel et al., 2011; Vocks et al., 2011). Most experiments, that examine reactions toward food, use pictures of food and ask ED women to rate their emotions on different dimensions (e.g., valence, arousal, pleasure, disgust, and fear) whilst viewing them (Rodríguez et al., 2007; Hay and Katsikitis, 2014), or to express their affects in an interview with an examiner (McNamara et al., 2008a). Although very informative, studies relying on self-reported measures could conduct to biases. First, social desirability and reluctance to provide information is one of the most important biases. In that sense, ED participants can soften, hide or alter the intensity of their true affects toward food for not seeming too disturbed or ill and, consequently, for avoiding the initiation or the prolongation of a treatment (Allison and Heshka, 1993; Nordbø et al., 2012; Brown et al., 2016). Second, high levels of alexithymia are found in individuals suffering from an ED (e.g., Schmidt et al., 1993; Gilboa-Schechtman et al., 2006; Nowakowski et al., 2013). Alexithymia is defined by difficulties identifying feelings and differentiating them from bodily sensations, difficulties describing and expressing emotions (i.e., lower emotional awareness), a lack of fantasy and a concrete cognitive style focused on the external environment (Sifneos, 1973; Taylor et al., 1991). Thus, the emotional responses that women with an ED give to food stimuli could be unprecise, unfelt or wrong. In the same vein, people suffering from AN or BN could report what they “cognitively think” of the stimuli instead of what they really “emotionally feel,” a phenomenon referred to the “cognitive-affective” division (Jenkins and O’Connor, 2012). Again, this phenomenon can reduce the exactness of women’s emotional responses toward food. To skirt these kinds of biases, indirect measures of emotions toward food are needed. Psychophysiological techniques (e.g., skin conductance, heat rate, startle reflex, cortisol level, electroencephalographic recordings) are an option. However, because these procedures are somewhat invasive and unpleasant - particularly for women with ED who are biologically monitored on a recurring basis – the development of a behavioral measure of emotions toward food would be useful.

Considering the facts that emotions influence timing and that time distortions (over- or underestimation of durations) give information about how the brain detects and interprets reality in terms of valence and arousal (Angrilli et al., 1997; Teixeira et al., 2013), time perception appears to be a sensitive way to explore emotional effects. In addition, if time perception tasks use food pictures as stimuli, then these tasks may become an innovative way for measuring and understanding emotions elicited in people with AN and BN. Therefore, the main objective of the present experiment was to assess the emotional impact provoked by food pictures in women suffering from an ED by using a temporal perception perspective, which allows to bypass the limits associated to traditional self-reported procedures. Because timing distortions caused by emotions seem to depend on the nature of the task used (Gil and Droit-Volet, 2011b), and that temporal processes present complexities that can be highlighted by specific paradigms (Baudouin et al., 2006; Mioni et al., 2013a,b, 2014; Ogden et al., 2014), two types of temporal tasks were selected for this investigation: a bisection and a duration discrimination tasks. According to the literature, the bisection task appears to be the most used method to study the effects of emotions on timing. Typically, this paradigm involves durations ranging from some milliseconds to seconds. The discrimination task, for its part, is a most classical method for investigating the mechanisms involved in the processing of short intervals (Grondin, 2008, 2010).

As ED are multifaceted psychiatric disorders, investigating emotions evoked by food stimuli in terms of time distortions requires supplementary considerations. One of those is the presence of affective comorbidities in women with ED, which can influence time perception. In fact, prevalence of depressive and anxiety symptoms is high among people suffering from AN and BN (Swinbourne et al., 2012; Aspen et al., 2014; Fakra et al., 2014; Godart et al., 2015; Meng and D’Arcy, 2015). Therewith, some studies showed that mood disturbances notably modulate time perception (for review, see Droit-Volet, 2013; Teixeira et al., 2013). Among them, Tipples (2008) found that negative emotionality was positively correlated to temporal bias due to angry and fearful expressions, and Mioni et al. (2016b) demonstrated that depressed patients over-produced durations, whereas anxious patients under-reproduced temporal intervals. Thus, in an experiment using temporal tasks with an ED population, the prior affective state of participants appears to be an important factor to take into account. Moreover, several researches have supported that women suffering from ED (or at higher risk) present cognitive difficulties (e.g., Lena et al., 2004; Steinglass and Glasofer, 2011; Jáuregui-Lobera, 2013; Weider et al., 2015; Naor-Ziv and Glicksohn, 2016) and, besides, it is well known that time perception relies on various cognitive processes (Perbal et al., 2002; Pouthas and Perbal, 2004; Zélanti and Droit-Volet, 2012). Even more important, cognitive abilities most often impaired in ED are those also involved in time processing, that is attention, processing speed, working memory, inhibition and switching (for ED, see Kemps et al., 2006; Rosval et al., 2006; Roberts et al., 2007; and for timing, see Zélanti and Droit-Volet, 2011; Mioni et al., 2012, 2013a,b; Pütz et al., 2012; Brown et al., 2013; Ogden et al., 2014; Droit-Volet et al., 2015). In consequence, for a better understanding of the performance of women with an ED in temporal tasks, it is crucial to document their cognitive abilities. Finally, because hunger modulates affective (valence, arousal), cerebral (activation, orientation of attention) and psychophysiological (salivation, heart rate) responses to food stimuli (Spence et al., 2016), can reduce cognitive effectiveness (Doniger et al., 2006; Benau et al., 2014) and is a key concept in ED symptomatology (Brown et al., 2010; Haedt-Matt and Keel, 2011), it appears essential to also get information about the level of appetite of participants during the experimentation. Keeping in mind all these considerations about the influence of prior affective state, cognitive abilities and appetite level on emotions and time processing, the second aim of the study was to identify factors that could contribute to explain the differences between participants’ performance on temporal tasks.

Globally, in accordance with Angrilli et al. (1997)’s point of view about the interaction between valence and arousal, we predicted that, for women suffering from an ED, food pictures will lead to a general overestimation of durations consecutive to an intense reaction of fear and an activation of the defensive system. Inversely, as found by Gil et al. (2009), we posited that for participants without ED, the duration of food pictures, even more the disgusting ones, will be underestimated due to a deviation of attentional resources from the passage of time. For this population, food pictures will not cause a strong reaction of fear and will not increase the arousal level. Finally, we anticipated that the performance of women with an ED on temporal tasks will be related to their clinical characteristics (i.e., BMI, ED symptomatology and affective state), level of hungriness and cognitive abilities (e.g., Tipples, 2008; Droit-Volet, 2013; Teixeira et al., 2013; Mioni et al., 2016b).

## Materials and Methods

### Participants

Twenty-three women suffering from an ED (i.e., ED group) and 23 healthy controls women (i.e., HC group) took part in the study. Participants were recruited among students and employees of Université Laval by electronic advertisements. For both groups, the inclusion criteria were the following: participants had to (a) be aged between 18 and 60 years; (b) be of French-Canadian origin; (c) be right-handed; (d) display normal or corrected-to-normal vision and audition; (e) be free of drug and alcohol abuses for 3 months; (f) not present a psychotic disorder; (g) not present a neurological disorder; and (g) show no history of traumatic brain injury within the last 5 years, or past head trauma associated with permanent cognitive impairments. For the HC group, additional criteria were used: (a) absence of personal or family ED antecedents; (b) no attempt to lose weight in the last month; and (c) absence of psychiatric disorder for which a medication was prescribed.

In accordance with the criteria of the *Diagnostic and Statistical Manual of Mental Disorders, Fifth Edition* (DSM-5; American Psychiatric Association, 2013), the ED group was composed of 10 women with AN (5 with restrictive subtype, 5 with binge eating/purging subtype) and 13 women with BN. The participants got their ED diagnostic from a health professional (i.e., psychiatrist, family doctor) a few weeks before the experiment started and still showed active symptoms during the study (*n* = 6), or from a doctoral-level psychologist (CG), which was then validated by a specialized clinical-researcher in the domain of evaluation and treatment of ED (CB; *n* = 17). All women suffering from an ED, if not yet engaged in a therapeutic process, were referred to clinical resources for support.

The participants of both groups gave informed written consent with respect to the Declaration of Helsinki. They received a monetary compensation of CAN $45 for their implication in the study. The experiment was approved by the Ethics Committee of the CHU de Québec – Université Laval (Project #2012-812, C11-08-088).

### Apparatus

For the temporal tasks, women were seated in front of a 16-inch CRT monitor connected to a PC, at a viewing distance of 60 cm. The room was dimly lit. The stimuli were presented in a 700 pixels × 526 pixels colored format, at the center of the screen, on a black font. The answers were collected by a keypad. The software E-Prime 2.0 Professional (Psychology Software Tools, Pittsburg, PA, United States, Released 2012) was used to create and administer the tasks. Except for the *Conners Continuous Performance Test – Second edition* (CPT-II, Conners, 2000), which was performed on a laptop, the neuropsychological tests were completed in a pencil-paper format. Statistical data analyses were performed with SPSS 24.0 for Windows (IBM Corporation, Released 2016) and the free software R 3.3.3 (R Core Team, Released 2017).

### Time Perception Tasks

#### Stimuli

**FIGURE 1:**
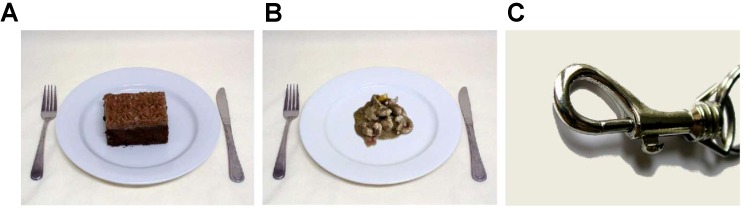
Examples of pictures used in the temporal tasks: **(A)** food eliciting joy (Web-FFQ; Labonté et al., 2012); **(B)** food eliciting disgust (Gagnon et al., 2018); **(C)** neutral objects (IAPS; Lang et al., 2008).

Two types of pictures were used in the temporal tasks: food pictures and object pictures. The food pictures were eliciting joy or disgust, and the object pictures were emotionally neutral. The stimuli were chosen based on data from a previous study (Gagnon et al., 2018), which aimed to identify, among women with ED, emotional responses to food pictures. Briefly, in this experiment, two groups of women, one with ED and one of HC, were asked to rate, on 9-point Self-Assessment Manikin (SAM; Lang, 1980; Bradley and Lang, 1994) or Likert scales (ranging from 1 to 9), 46 food and 12 object pictures on nine dimensions (i.e., valence and arousal on SAM scales; joy, sadness, anger, disgust, fear, surprise and neutrality on Likert scales). From this collection of pictures, the five food pictures that generated the highest level of joy, the five food pictures that evoked the highest level of disgust, and the five object pictures that were the most neutral – on Likert scales of joy, disgust and neutrality, respectively – were chosen for the present study. The top-5 pictures needed also to evocate, for the joyful ones, a positive valence (>5 value), for the disgusting ones, a negative valence (<5 value), and for the neutral object pictures, an intermediate valence (value between 4 and 6). Because pictures in the top-5 food images that provoked joy in women with ED were not all the same that those for HC, some joyful pictures used for the two groups were different. In fact, as the main utility of the pictures was to elicit strong emotions, their selection was based on the intensity of their targeted affect instead of the similarity of their nature. Inversely, the top-5 disgusting food and the top-5 neutral object pictures were identical for both groups, so the temporal tasks adopted the same pictures for women with ED and HC. Ultimately, the selected joyful food pictures for the ED group were: strawberries, pieces of pineapple, red grapes, mixed salad leaves and a crepe. Those for HC were: strawberries, pieces of pineapple, a slice of sugar pie, squares of chocolate bar and a piece of chocolate cake. For both groups, the selected disgusting food pictures were: a black blood sausage, winkles, pieces of chitterlings sausage, a black radish and pieces of kidney in sauce. Finally, the neutral objects for all women were: a snap hook, a lamp, a wall socket, pincers and a screw. **Table 1** shows values of selected pictures on emotional dimensions that are interesting for the present study, and **Figure 1** shows examples of those pictures.

**Table 1:** Values of selected pictures on valence, arousal, joy, disgust and neutrality.

	Emotional dimensions				
	
Pictures	Valence	Arousal	Joy	Disgust	Neutrality
Black blood sausage	2.32 (1.81)^a^	6.41 (2.40)	1.18 (0.66)	8.05 (1.84)	2.23 (1.69)
	2.64 (1.26)^b^	4.50 (2.63)	1.27 (0.88)	6.73 (2.39)	2.59 (2.58)
Black radish	2.64 (1.56)	5.50 (2.06)	1.05 (0.21)	7.36 (2.08)	2.86 (2.44)
	3.09 (1.63)	4.68 (2.25)	1.18 (0.50)	6.14 (2.82)	3.27 (2.76)
Crepe	5.82 (2.20)	6.05 (2.13)	4.91 (2.67)	2.09 (1.93)	2.82 (2.04)
	7.64 (0.95)	6.86 (1.75)	6.55 (2.40)	1.00 (0.00)	2.27 (1.88)
Lamp	4.95 (0.95)	2.82 (1.84)	1.41 (1.05)	1.18 (0.59)	7.14 (2.46)
	4.41 (1.22)	2.95 (1.89)	1.45 (1.01)	1.27 (1.28)	7.27 (2.96)
Mixed salad leaves	6.77 (2.16)	3.86 (3.08)	5.05 (2.89)	1.32 (1.29)	3.91 (3.08)
	6.05 (1.53)	5.18 (1.79)	4.36 (2.50)	1.14 (0.64)	4.00 (2.49)
Pieces of chitterlings sausage	3.05 (1.84)	5.32 (2.32)	1.32 (0.57)	7.45 (2.24)	3.36 (2.80)
	3.32 (1.36)	4.45 (2.32)	1.23 (0.69)	5.68 (2.75)	3.27 (2.60)
Piece of chocolate cake	4.77 (2.83)	7.14 (1.86)	3.95 (3.12)	3.73 (3.45)	1.82 (1.40)
	7.68 (1.29)	7.09 (1.69)	6.82 (2.32)	1.32 (0.95)	2.18 (1.59)
Pieces of kidney in sauce	2.27 (1.35)	5.64 (2.13)	1.23 (0.53)	7.18 (2.15)	3.05 (2.13)
	3.18 (1.68)	5.09 (1.97)	1.86 (1.73)	5.91 (2.43)	2.68 (2.68)
Pieces of pineapple	7.50 (1.77)	6.00 (1.88)	6.64 (2.52)	1.00 (0.00)	2.77 (2.33)
	8.14 (0.83)	7.59 (1.40)	7.59 (1.92)	1.00 (0.00)	2.05 (1.43)
Pincers	5.00 (0.69)	2.86 (2.01)	1.55 (1.50)	1.14 (0.64)	7.09 (2.62)
	4.36 (1.36)	3.59 (1.84)	1.27 (0.88)	1.09 (0.43)	6.59 (3.26)
Red grapes	7.59 (1.53)	5.41 (2.36)	6.55 (2.36)	1.05 (0.21)	3.50 (2.41)
	7.27 (1.24)	6.18 (2.22)	6.32 (2.08)	1.05 (0.21)	2.55 (1.99)
Screw	4.55 (1.26)	2.86 (1.83)	1.32 (1.49)	1.23 (1.07)	7.55 (2.06)
	4.23 (1.23)	3.50 (2.02)	1.41 (1.01)	1.45 (1.06)	6.14 (3.28)
Slice of sugar pie	3.64 (2.75)	6.91 (2.33)	3.05 (2.59)	4.77 (3.48)	2.09 (1.63)
	8.09 (1.11)	7.59 (1.71)	7.36 (1.89)	1.09 (0.29)	1.95 (1.81)
Snap hook	4.86 (0.99)	2.77 (2.07)	1.68 (1.67)	1.05 (0.21)	7.27 (2.51)
	4.45 (1.44)	3.23 (2.09)	1.45 (1.01)	1.00 (0.00)	7.64 (2.48)
Squares of chocolate bar	4.59 (3.11)	6.91 (1.90)	4.27 (3.06)	3.32 (3.33)	1.95 (1.40)
	8.00 (1.38)	7.50 (1.44)	7.27 (1.93)	1.14 (0.47)	2.00 (1.51)
Strawberries	8.00 (1.20)	5.77 (2.41)	6.95 (1.84)	1.00 (0.00)	2.73 (1.96)
	8.45 (0.91)	7.82 (1.47)	7.73 (1.78)	1.00 (0.00)	1.64 (1.18)
Wall socket	4.91 (0.97)	2.41 (1.82)	1.27 (0.77)	1.09 (0.43)	7.82 (2.06)
	4.68 (1.04)	3.32 (2.03)	1.77 (1.27)	1.14 (0.64)	6.95 (2.40)
Winkles	2.82 (1.68)	5.68 (2.12)	1.27 (0.88)	7.77 (2.05)	2.59 (2.02)
	2.73 (1.16)	4.82 (2.28)	1.09 (0.29)	6.45 (2.50)	2.68 (2.68)


*Values represent means (standard deviations) on Likert scales ranging from 1 to 9. ^*a*^Eating disorders group. ^*b*^Healthy controls group.*

All joyful food pictures came from the Web Food Frequency Questionnaire (Web-FFQ; Labonté et al., 2012), while disgusting food pictures were created with copyright free pictures found on the Internet and stimuli used by Rousset et al. (2005, 2008). The visual parameters of these pictures were standardized (e.g., resolution, font, frame, brilliance) and the food was always presented in the same way, that is on the center of a white plate, bordered by a knife and a fork. Also, the size of the food portion on the dish (i.e., area covered by the aliment or the meal) was similar across pictures. Finally, the object pictures were extracted from the International Affective Picture System (IAPS, #7059, 7175, 6150, 7056, 7018; Lang et al., 2008).

#### Temporal Bisection Tasks

Inspired by the research of Gil et al. (2009) on time perception and food pictures, the study was composed of two bisection tasks: one with emotional pictures (i.e., joyful and disgusting food), and one with a neutral picture. However, instead of using a white oval as a non-food or neutral stimulus like precedent authors, here, pictures of a neutral object were exploited. In fact, for women suffering from an ED, a white oval could represent an empty plate, which, according to clinical practice, seems far from being emotionally neutral.

Each bisection task consisted of two phases: a training and a testing phases. During the training phase, women were exposed to a short (400 ms) and to a long (1600 ms) standard durations, which were marked by the presentation of a picture of a snap hook. This object was chosen because, as said previously, it was evaluated as emotionally neutral by both groups, and in order to make the visual parameters of images uniform, its size was comparable to the size of food used as emotional stimuli. The S and L standard durations were presented five times each, and the women had to memorize them. After that, the participants performed 14 practice trials (2 for each probe duration) in which they had to indicate, by pressing the corresponding button (S or L) on the keypad, whether the presentation duration of a new picture, a screw, was closer to the short (S) or to the long (L) standard duration. As for the snap hook, the picture of the screw was chosen for its neutrality and its size. In order for the emotional salience of the stimuli to take hold, a delay of 1800 ms separated the duration of the stimuli to be judged and the screen asking participants to give their answer. The left–right position of keys was counterbalanced across women. Seven probe durations were used: the two standards (400 and 1600 ms) and five intermediate duration values (600, 800, 1000, 1200, and 1400 ms). A retroaction was given for every trial of the training phase. The intertrial interval (ITI) was then presented, with a random duration ranging from 1800 to 2300 ms.

During the testing phase, the women performed the same task: they indicated whether the presentation duration of a picture was closer to the S or to the L standard duration. However, whereas the stimuli took the form of an object picture (snap hook) in the neutral bisection task, food pictures were used in the emotional conditions. In the neutral task, each participant completed a total of 280 trials, that is 40 trials for each probe duration (40 × 7), separated into 5 blocks of 56 trials. In the emotional task with food pictures, women performed 560 trials, that is 40 trials for each probe duration, for each emotion (40 × 7 × 2)^1^. These 560 trials were divided in 8 blocks of 70 trials. As mentioned above, five specific joyful stimuli were used for ED and HC groups, but both groups viewed the same five images of disgusting food. The use of five different images to evoke the same affect was motivated by a desire to reduce emotional habituation across blocks and trials. In this sense, for example, instead of viewing a joyful food picture 280 times, participants saw it 56 times.

In the two bisection tasks, trials were presented in a random order. The standards were showed at the start of each block, before the round of trials began. A break was taken by the participants between the blocks to reduce fatigue. Women were asked to refrain from using segmentation and counting strategies that could help them to track time (e.g., foot taping, imaging, repetitive movements, or counting seconds; Grondin et al., 1999).

#### Duration Discrimination Task

A discrimination task was also proposed to participants. In this task, women of both groups had to judge the relative duration of two pictures presented successively and to indicate, by pressing the appropriate button on the keypad (S or L), whether the duration of the second picture was shorter (S) or longer (L) than the first. The left-right position of keys was counterbalanced across participants. The images were presented back to back, in a random order, with a 1 s interstimulus interval (ISI). The participants had to respond as soon as the second picture disappeared of the screen, which then turned black: there were neither a fixed delay between the second stimulus and the response, nor a screen of instructions asking participants to enter their answer. No training phase was introduced before the testing blocks and no feedback was given after each response.

The two stimuli presented consecutively could be of three emotions: joy (J), disgust (D), or neutral (N). So, nine conditions composed the task: three in which the same emotion was used to mark the duration of the first and second pictures (J–J, D–D, N–N), and six conditions in which different emotions were used (J–D, J–N, D–N, D–J, N–J, N–D). There was one experimental block for each of these nine combinations. The order of the blocks was randomized and counterbalanced between participants. There was a total of 360 trials, that is 40 trials for each of the 9 blocks. Durations used were 400 and 482 ms. Within each block, the order of the short and the long intervals was randomized, but equiprobable. As for the bisection tasks, one emotion was solicited by five different images, but contrary to that task, the neutral affect was also represented by five pictures. Again, each group tested specific pictures for the joyful food conditions, but were presented with the same pictures for the disgusting ones. The neutral object pictures were also identical for the ED and the HC groups.

### Measures

#### Anthropometric Data

Participants’ height and weight were measured, then their body mass index (BMI; kg/m^2^) was calculated. The direct evaluation of anthropometric data was made instead of using self-reported information because the latter can be invalid (Meyer et al., 2009a,b; Ambwani and Chmielewski, 2013).

#### ED Symptomatology

*The Eating Disorder Examination – Questionnaire* (EDE-Q 6.0; Fairburn and Beglin, 2008; French translation by Carrard et al., 2015) and the *Revised Restraint Scale* (RRS; Herman and Polivy, 1980; unofficial French translation by the Institut sur la Nutrition et les Aliments Fonctionnels [INAF] of Université Laval), two self-report scales, were used to confirm the absence of ED in the HC group, and to document the ED symptomatology in the clinical group. The EDE-Q includes 22 items assessing the attitudinal features of ED psychopathology, which can be derived in four subscales: restraint, eating concern, shape concern and weight concern over the 28-previous day. These items are answered on a 7-point Likert scale (ranging from 0 to 6). A global score can be calculated by summing and averaging the subscales scores. The greater the global score is, the more severe are the symptoms of ED. Another six items assess the frequency of ED behaviors (i.e., binge eating episodes, inappropriate compensatory methods), that is how many times the behaviors occurred during the 28-previous days. The EDE-Q is a good instrument in terms of internal consistency (α = 0.70–0.90 for clinical sample; α = 0.78–0.93 for community sample; Luce and Crowther, 1999; Mond et al., 2004b; Peterson et al., 2007) and test–retest reliability (*r* = 0.81–0.94 for 2 weeks, *r* = 0.57–0.77 for about 1 year; Luce and Crowther, 1999; Mond et al., 2004a). To be included in the study as HC, recruited women could not present any fasting phase or notable restriction of energy intake to lose weight, nor any episode of binge eating with inappropriate compensatory behaviors. Furthermore, HC participants had to be satisfied with their weight and silhouette (dissatisfaction score < 3 on both items), and these elements could not influence significantly their self-esteem (score < 3).

The RRS measures restrained eating. It has 10 items divided into two subscales: concern for dieting and weight fluctuation. These items are presented in a multiple-choice format and scored on scales of 0–3 or 0–4 points. A typical cut-off score of 15 or 16 is used to designate high restraint (Polivy et al., 1988; Heatherton et al., 1991), so to take part in the study, recruited HC should get a global result inferior or equal to 15. The RRS has good psychometric proprieties: internal consistency ranges from 0.79 to 0.86, and test–retest reliability is evaluated to 0.95 for 2 weeks and 0.74 for 2 years (Ruderman, 1983; Allison et al., 1992; Gorman and Allison, 1995).

In addition to the EDE-Q and the RRS, the *Structured Clinical Interview for DSM-IV-TR Axis I Disorders – Research version, Patient Edition* (SCID-I/P; First et al., 2002), adapted for the DSM-5 criteria (American Psychiatric Association, 2013), was used to attribute diagnoses to women in the ED group. The SCID-I/P is a gold standard semi-structured interview for psychiatric evaluation and diagnostic. The instrument has an interjudge reliability of 0.70–1.0 and a test–retest reliability of 0.82–0.90 for both clinical and community samples (Williams et al., 1992; Segal et al., 1994; Pike et al., 1995; First et al., 2002).

#### Affective State

Depressive symptoms of women in both groups were evaluated with the *Beck Depression Inventory II* (BDI-II; Beck et al., 1996; French adaptation by Éditions du Centre de Psychologie Appliquée, 1996). This questionnaire has 21 items with ratings ranging from 0 to 3, according to the severity of symptoms. Participants must respond as a function of their state during the last 2 weeks, including the day of testing. The BDI-II global score estimates the intensity of the depressive mood, with higher scores indicating more severe symptoms. The instrument has a high internal consistency (α = 0.90) and a good test–retest stability (*r* = 0.73–0.96; for review, see Wang and Gorenstein, 2013).

The *State-Trait Anxiety Inventory – Y Form* (STAI-Y, Spielberger, 1983; French-Canadian adaptation by Gauthier and Bouchard, 1993), a 40-item self-questionnaire, was used to detect the presence and assess the intensity of anxiety symptoms in participants. Part A assesses State-Anxiety, which can be defined as subjective feelings of tension, nervousness and worry, and the arousal of the autonomic nervous system induced temporarily by situations perceived as dangerous. At the opposite, Part B assesses Trait-Anxiety, which refers to a tendency to perceive the world in a fearful way, and to a disposition to react toward it with stress and discomfort in a predictable manner. Part A consists of 20 statements evaluating how respondents feel “right now” or “in a recent past” according to the moment they complete the questionnaire, whereas Part B consists of 20 items assessing how people “generally” feel. Participants respond on 4-point Likert scales. Two raw scores, one for each part, are obtained by adding up the points on each item, and are then transformed into *T*-scores depending on the age of the participants. For the needs of the experiment, only Part A scores (i.e., actual symptoms; State-Anxiety) were compiled. Test–retest reliability coefficient of the instrument ranges from 0.31 to 0.86 (intervals fluctuating from 1 h to 104 days) and its internal consistency coefficient ranges from 0.86 to 0.95 (Spielberger, 1983; Gauthier and Bouchard, 1993).

A supplementary measure of participants’ affective state was used: the *Profile of Mood States* (POMS; McNair et al., 1971; French adaptation by Cayrou et al., 2000, 2003). This 65-item questionnaire was introduced in the study to consider feelings other than depression and anxiety, and to collect data about the mood of participants just before they complete temporal tasks (i.e., their actual or “right now” mood). In fact, the POMS provides a score for different emotional conditions (i.e., tension-anxiety, depression, anger-hostility, vigor-activity, fatigue, confusion-bewilderment, and interpersonal relationships), and a total mood disturbances score (TMD) for which all scales except interpersonal relationships are taken into account. For each item, respondents give a self-reported answer on how they do or do not relate. Five-point Likert scales are used. Raw scores are transformed into *Z*-scores, then into *T*-scores. For the present study, only the TMD score, a general distress estimate, was considered. The POMS has satisfying psychometric qualities: its internal consistency ranges from 0.63 to 0.88 and its 2-week test–retest reliability ranges from 0.66 to 0.83 (McNair et al., 1971; Cayrou et al., 2000, 2003).

#### Cognitive Abilities

Five well-known neuropsychological tests were used to evaluate attention components, processing speed, working memory, and executive functioning of all participants. The *Continuous Performance Test – Second Edition* (CPT-II; Conners, 2000) is a computerized task that requires respondents to press the spacebar each time a letter appears on the screen, except when the letter X is shown. The 14-min duration of the test and the variation of its ISI allow measuring sustained attention and vigilance. Alertness level (reaction time) and its constancy (or stability) are also evaluated, as well as the number of commissions (i.e., number of times the spacebar is pressed when the letter X is shown) and omissions (i.e., number of letters for which no response is provided). Performance is expressed in *T*-scores: higher are the *T*-scores, greater are the participants’ difficulties.

The *Spatial Span* subtest of the *Wechsler Memory Scale – Third Edition* (WMS-III; Wechsler, 1997) assesses non-verbal (or visuospatial) working memory (i.e., maintenance and manipulation components). Ten blocks are arranged asymmetrically on a board. The examiner taps the blocks in a prearranged sequence and the participant tries to reproduce it by pointing the blocks in the same (Spatial Span Forward; maintenance component) or in the reverse order (Spatial Span Backward; manipulation component), as instructed. The number of blocks to be recalled increases across trials. The task is discontinued when the respondent commits two errors in both trials of the same length. The span represents the number of blocks tapped in the longest set completed with success. The performance is expressed in cumulative percentages: higher are the scores, lower are the participant’s spans.

Two tasks of the *Delis-Kaplan Executive Function System* (D-KEFS; Delis et al., 2001) were used to assess women’s performance in visual scanning (i.e., selective attention), processing speed, verbal inhibition, and cognitive flexibility. The first one is the *Trail Making Test* (TMT), which is composed of five parts. In Part 1 (visual scanning), respondents are required to find and circle all the numbers “3” on a worksheet with distractors (numbers and letters). In Part 2 (number sequencing), participants must link a set of 16 numbers (1 to 16) in ascending order, on a worksheet with numbers and letters. In Part 3 (letter sequencing), they have to join a set of letters (A to P) in alphabetical order. In Part 4 (number-letter switching), respondents must connect 16 numbers and 16 letters, in numerical and alphabetical orders, in switching between them (i.e., 1-A-2-B-3-C). Finally, in Part 5 (motor speed), women have to draw a line over a dotted line, in touching circles along the path, as fast as they can. For every part, the time to complete the task and the number of errors are recorded. The performance is expressed in scaled-scores: higher are the scaled-scores, better are the participants’ abilities. The second task of the D-KEFS that was used is the *Color-Word Interference* (CWI). This subtest, based on the Stroop effect, has four conditions. In Condition 1 (denomination), respondents name the color of squares printed on a sheet. In Condition 2 (reading), women read color names (words) printed in black ink. In Condition 3 (inhibition), participants have to name the color of the ink in which given color words are printed, that is to inhibit automatic responses (i.e., reading) and generate incongruent responses. In Condition 4 (switching), participants must shift between reading the color names (words) and naming the ink colors of the words printed. Like the TMT, for all conditions, time to achieve the task and number of errors are noted. Once again, the performance is expressed in scaled-scores.

Finally, participants’ verbal working memory (i.e., maintenance and manipulation components) was assessed by the *Digit Span* subtest of the *Wechsler Adult Intelligence Scale – Fourth Edition: Canadian* (WAIS-IV CDN; Wechsler, 2008). In this task, the examiner verbalizes a sequence of numbers and the respondent is asked to repeat them in the same order (Digit Span Forward; maintenance component), in reverse order (Digit Span Backward; manipulation component) or in ascending order (Digit Span Sequencing; manipulation component), as instructed. As for the *Spatial Span*, the task is discontinued when the participant commits two errors in both trials of the same length, and the span represents the number of digits recalled in the longest set correctly completed. The performance is expressed in cumulative percentages: higher are the scores, lower are the participant’s spans.

### Procedure

Women of both groups were tested individually in a quiet room of the Laboratoire de Recherche en Psychologie de la Perception of the Université Laval. To reduce the variability of hungriness between participants and its influence on time perception, women were told to eat in the 60 min preceding the experiment and were tested at fixed hours, that is after breakfast (8:30 am), after lunch (1:30 pm) or after supper (6:30 pm). The study included two sessions that lasted approximatively 2.5 h each. At the beginning of each session, participants had to relate the time and the content of their preceding meal, evaluate their level of appetite on a 7-point Likert scale (ranging from 0 to 6) and fill in the POMS about their actual general mood. During the first experimental session, women completed the emotional bisection task, filled in questionnaires about their eating behaviors and affective state (i.e., EDE-Q, RRS, BDI-II, and STAI-Y), and achieved half of the neuropsychological measures. During the second session, they performed the neutral bisection task, finished the neuropsychological testing and realized the discrimination task. Afterwards, anthropometrics data were collected. The order of the sessions and the content of each seance were counterbalanced across participants. Considering the cognitive load linked to each experimental seance, a large break was taken between the tasks and only one session could be achieved per day. However, to reduce the variability of affective state and BMI across seance, both sessions had to be completed inside 2 weeks.

## Results

### Age, BMI, ED Symptomatology, Affective State and Level of Hungriness

Demographic information, clinical characteristics and level of hungriness of participants are reported in **Table 2**. Values for each group of women (i.e., ED and HC), and for each ED subgroup (i.e., AN and BN) are presented. Data were inspected for normality (skewness, kurtosis, Shapiro–Wilk test), and because they did not display a normal distribution, non-parametric analyses (i.e., Kruskal–Wallis and Mann–Whitney *U* tests) were used to evaluate differences between groups and subgroups. The alpha (0.05) was adjusted with a Bonferroni correction when needed. **Table 3** provides the results of the analyses.

**Table 2:** Demographic information, clinical characteristics, and level of hungriness for groups of eating disorders (ED) and healthy controls (HC), and for anorexia (AN) and bulimia nervosa (BN) subgroups of ED.

	ED group	HC group	AN subgroup	BN subgroup
Variable	*M (SD)*	*M (SD)*	*M (SD)*	*M (SD)*
Age (years)	30.35 (11.31)	25.91 (5.86)	30.80 (13.32)	30.00 (10.06)
BMI (kg/m^2^)	22.92 (4.56)	21.47 (2.17)	19.54 (2.21)	25.53 (4.20)
EDE-Q	3.47 (0.99)	0.59 (0.45)	3.56 (0.96)	3.39 (1.04)
RRS	22.61 (3.09)	8.83 (4.02)	21.10 (2.85)	23.77 (2.83)
BDI-II	13.43 (10.97)	3.13 (2.69)	19.80 (8.12)	8.54 (10.56)
STAI-Y (part A)	56.09 (10.79)	43.70 (4.79)	62.30 (8.58)	51.31 (10.07)
POMS _Bisection_	41.59 (7.98)	35.12 (1.92)	45.67 (7.87)	38.45 (6.77)
POMS _Discrimination_	41.69 (8.66)	35.42 (2.86)	45.75 (9.68)	38.56 (6.56)
Hungriness _Bisection_	1.01 (0.93)	0.67 (0.73)	0.58 (0.71)	1.35 (0.97)
Hungriness _Discrimination_	1.20 (1.32)	0.87 (1.10)	0.85 (1.38)	1.46 (1.27)


*BMI, Body mass index; EDE-Q, Eating Disorder Examination – Questionnaire (global score); RRS, Revised Restraint Scale (raw score); BDI-II, Beck Depression Inventory II (raw score); STAI-Y, State-Trait Anxiety Inventory – Y Form (T-score); POMS, Profile of Mood State (Global Mood Disturbances T-score); Hungriness, Level of appetite assessed on a 7-point Likert scale ranging from 0 to 6.*

**Table 3:** Results of the Mann–Whitney *U* and the Kruskal–Wallis tests on demographic information, clinical characteristics and level of hungriness of participants.

	ED vs. HC		AN vs. BN vs. HC	
		
Variable	*U*	*R*_b_	*H*(2, *N* = 46)	*E*^2^_H_
Age (years)	220.00	0.17	1.35	0.03
BMI (kg/m^2^)	233.00	0.12	16.49^∗^	0.37
EDE-Q	2.00^∗^	0.99	33.29^∗^	0.74
RRS	0.50^∗^	0.99	34.78^∗^	0.77
BDI-II	70.50^∗^	0.73	24.30^∗^	0.54
STAI-Y (part A)	59.50^∗^	0.78	24.49^∗^	0.54
POMS _Bisection_	95.50^∗^	0.64	18.81^∗^	0.42
POMS _Discrimination_	108.50^∗^	0.59	16.37^∗^	0.36
Hungriness _Bisection_	202.00	0.24	34.75^∗^	0.77
Hungriness _Discrimination_	227.00	0.14	3.04	0.07


*ED, eating disorders group; HC, healthy controls group; AN, anorexia nervosa subgroup; BN, bulimia nervosa subgroup; BMI, body mass index; EDE-Q, Eating Disorder Examination – Questionnaire (global score); RRS, Revised Restraint Scale (raw score); BDI-II, Beck Depression Inventory II (raw score); STAI-Y, State-Trait Anxiety Inventory – Y Form (T-score); POMS, Profile of Mood State (Global Mood Disturbances T-score); Hungriness, level of appetite assessed on a 7-point Likert scale ranging from 0 to 6. ^∗^Significant effect (alpha level adjusted with a Bonferroni correction when needed).*

At the groups level, there were no significant differences between women with ED and HC for age (*p* = 0.327), BMI (*p* = 0.489) and level of hungriness before each experimental session (for bisection, *p* = 0.159; for discrimination, *p* = 0.382). However, women suffering from AN or BN as a group showed more attitudinal ED features (*p* < 0.001), restrained eating (*p* < 0.001), depression symptoms (*p* < 0.001), anxiety manifestations (*p* < 0.001), and global mood disturbances for both experimental seances (for bisection, *p* < 0.001; for discrimination, *p* = 0.001) than HC.

At the subgroups level, there were no significant differences between women with AN, BN and HC for age (*p* = 0.509) and level of hungriness before the discrimination task (*p* = 0.219). However, there were differences between subgroups on the BMI (*p* < 0.001), the BDI-II (*p* < 0.001), the STAI-Y (*p* < 0.001), the EDE-Q (*p* < 0.001), the RRS (*p* < 0.001), the POMS for both sessions (for bisection, *p* < 0.001; for discrimination, *p* < 0.001) and the level of hungriness before the bisection task (*p* < 0.001). More precisely, the BN subgroup showed a higher BMI than the AN subgroup (*p* < 0.001) and the HC group (*p* = 0.021), and a higher level of hungriness during the bisection task than the HC group (*p* = 0.042). In addition, this subgroup presented lower symptoms of depression than women suffering from AN (*p* = 0.044), but higher levels of anxiety (*p* = 0.017), ED attitudes (*p* < 0.001) and retrained eating (*p* < 0.001) than HC. For their part, women with AN showed a higher level of depression (*p* < 0.001), anxiety (*p* < 0.001), ED features (*p* < 0.001) and restrained eating (*p* < 0.001) than HC. They showed also a higher level of general distress than HC in the beginning of each experimental session (*p* < 0.001).

### Cognitive Abilities

**Table 4** presents the classification of neuropsychological measures according to the cognitive domain assessed, and the results on these measures. Data for each group of women, and for each ED subgroup are shown. To verify the presence of differences between groups and subgroups, non-parametric analyses were used once again because the data were not normally distributed. Each cognitive domain was evaluated separately and the alpha (0.05) was adjusted when requisite (see **Table 5**).

**Table 4:** Scores on neuropsychological measures, by cognitive domain, for groups of eating disorders (ED) and healthy controls (HC), and for anorexia (AN) and bulimia nervosa (BN) subgroups of ED.

		ED group	HC group	AN subgroup	BN subgroup
Cognitive domain	Measure	*M (SD)*	*M (SD)*	*M (SD)*	*M (SD)*
Working memory – Maintenance	Spatial span _Forward_^a,b^	77.74 (21.74)	71.96 (16.51)	71.65 (24.47)	82.42 (19.05)
	Digit span _Forward_^a,c^	63.32 (30.32)	53.43 (27.00)	79.01 (19.11)	51.25 (32.38)
Working memory – Manipulation	Spatial span _Backward_^a,b^	45.63 (24.73)	45.70 (21.60)	45.70 (20.49)	45.58 (28.40)
	Digit span _Backward_^a,c^	64.13 (33.97)	53.48 (31.53)	78.85 (21.88)	52.81 (37.90)
	Digit span _Sequencing_^a,c^	62.46 (31.29)	61.82 (31.30)	60.70 (38.86)	63.81 (25.64)
Processing speed	CWI _Part 2_	11.70 (1.22)	12.43 (1.56)	11.80 (1.03)	11.62 (1.39)
Alertness	CPT-II _Hit RT_	46.00 (8.78)	47.47 (12.83)	44.70 (8.41)	47.00 (9.27)
	CPT-II _Hit RT SD_	46.75 (10.26)	39.85 (13.72)	47.56 (11.17)	46.13 (9.93)
Selective attention	TMT _Part 1_	11.65 (1.27)	12.30 (1.15)	11.70 (0.82)	11.62 (1.56)
Sustained attention	CPT-II _Hit RT Block Change_	45.31 (9.05)	48.19 (9.52)	47.18 (12.02)	43.87 (6.05)
	CPT-II _Omissions_	47.87 (4.31)	46.37 (3.34)	46.97 (3.70)	48.56 (4.75)
Vigilance	CPT _Hit RT ISI Change_	52.02 (9.26)	45.50 (9.26)	54.33 (8.42)	50.25 (9.81)
Motor inhibition	CPT-II _Commissions_	54.45 (11.65)	46.74 (8.95)	57.77 (13.10)	51.89 (10.20)
Verbal inhibition	CWI _Part 3 vs. *Part* 1_	11.00 (1.04)	11.09 (1.62)	11.00 (0.94)	11.00 (1.15)
Cognitive flexibility	CWI _Part 4 vs. *Part* 1 + Part 2_	10.17 (1.67)	10.04 (1.33)	10.30 (1.70)	10.08 (1.71)
	TMT _Part 4 vs. *Part* 2 + Part 3_	10.22 (2.15)	9.39 (1.62)	10.60 (2.59)	9.92 (1.80)


*CPT-II, Continuous Performance Test – Second Edition; RT, reaction time; SD, standard deviation; TMT, Trail Making Test; BC, block change; ISI, interstimulus interval. Types of measures for the neuropsychological tests: Spatial Span and Digit Span, cumulative percentages; CPT-II, T-scores; CWI and TMT, scaled scores. ^*a*^Longest sequence correctly recalled (span); ^*b*^non-verbal modality; ^*c*^verbal modality.*

**Table 5:** Results of the Mann–Whitney *U* and the Kruskal–Wallis tests on participants’ neuropsychological scores, by cognitive domain.

		ED vs. HC		AN vs. BN vs. HC	
			
Cognitive domain	Measure	*U*	*R*_b_	*H*(2, *N* = 46)	*E*^2^_H_
Working memory – Maintenance	Spatial span _Forward_^a,b^	198.50	0.25	3.35	0.07
	Digit span _Forward_^a,c^	218.00	0.18	6.58^∗^	0.15
Working memory – Manipulation	Spatial span _Backward_^a,b^	280.00	0.06	0.19	<0.01
	Digit span _Backward_^a,c^	217.00	0.18	4.76	0.11
	Digit span _Sequencing_^a,c^	264.00	<0.01	0.11	<0.01
Processing speed	CWI _Part 2_	351.00^∗^	0.33	3.92	0.09
Alertness	CPT-II _Hit RT_	258.00	0.02	0.30	<0.01
	CPT-II _Hit RT SD_	162.00^∗^	0.39	5.20	0.12
Selective attention	TMT _Part 1_	346.50^†^	0.31	3.55	0.08
Sustained attention	CPT-II _Hit RT Block Change_	306.00	0.16	3.29	0.07
	CPT-II _Omissions_	193.00	0.27	3.72	0.08
Vigilance	CPT _Hit RT ISI Change_	147.00^∗^	0.44	7.49^∗^	0.17
Motor inhibition	CPT-II _Commissions_	163.50^∗^	0.38	5.95	0.13
Verbal inhibition	CWI _Part 3 vs. Part 1_	276.50	0.05	0.09	<0.01
Cognitive flexibility	CWI _Part 4 vs. Part 1 + Part 2_	261.00	0.01	0.15	<0.01
	TMT _Part 4 vs. Part 2 + Part 3_	222.00	0.16	2.32	0.05


*ED, eating disorders group; HC, healthy controls group; AN, anorexia nervosa subgroup; BN, bulimia nervosa subgroup; CPT-II, Continuous Performance Test – Second Edition; RT, reaction time; SD, standard deviation; TMT, Trail Making Test; BC, block change; ISI, interstimulus interval. Types of measures for the neuropsychological tests: Spatial Span and Digit Span, cumulative percentages; CPT-II, T-scores; CWI and TMT, scaled scores. ^*a*^Longest sequence correctly recalled (span); ^*b*^non-verbal modality; ^*c*^verbal modality. ^∗^Significant effect (alpha level adjusted with a Bonferroni correction when needed). ^†^Marginally significant effect (alpha level adjusted with a Bonferroni correction when needed).*

At the groups level, the analyses revealed that women with an ED showed verbal (maintenance component, *p* = 0.303; manipulation component, *p* = 0.295, *p* = 0.991) and visuospatial (maintenance component, *p* = 0.143; manipulation component, *p* = 0.732) working memory, reaction time (*p* = 0.886), sustained attention (*p* = 0.362, *p* = 0.101), verbal inhibition (*p* = 0.786) and cognitive flexibility abilities (*p* = 0.936, *p* = 0.328) similar to those of the HC group. Nevertheless, their processing speed was slower (*p* = 0.049), their alertness was less constant (*p* = 0.024), their vigilance was inferior (*p* = 0.010), and their motor impulsivity (*p* = 0.026) was higher than those of HC. Furthermore, the ED group tended to have lower selective attention (visual scanning) capacities than women not suffering from AN or BN as a group (*p* = 0.062).

At the subgroups level, there were no significant differences between women for the manipulation component in verbal (*p* = 0.092, *p* = 0.949) and visuospatial (*p* = 0.908) working memory, the maintenance component in spatial working memory (*p* = 0.187), the processing speed (*p* = 0.141), the alertness (level, *p* = 0.861; stability, *p* = 0.074), the selective attention (*p* = 0.170), the sustained attention (*p* = 0.193, *p* = 0.156), the motor impulsivity (*p* = 0.051), the verbal inhibition (*p* = 0.955), and the cognitive flexibility (*p* = 0.928, *p* = 0.313). However, the vigilance capacities of the AN subgroup were poorer than those of the HC group (*p* = 0.030). In addition, women suffering from AN tended to show a lower verbal span for the maintenance condition than the BN (*p* = 0.057) and HC participants (*p* = 0.069).

### Performance on Time Perception Tasks

#### Temporal Bisection Tasks

**FIGURE 2:**
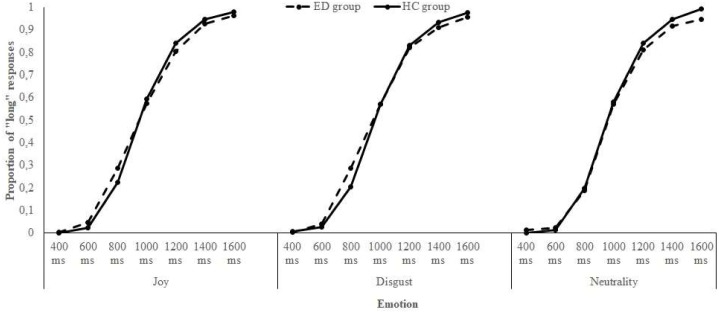
Proportion of “long” responses plotted against stimulus duration for each emotional condition and each group of participants.

**FIGURE 3:**
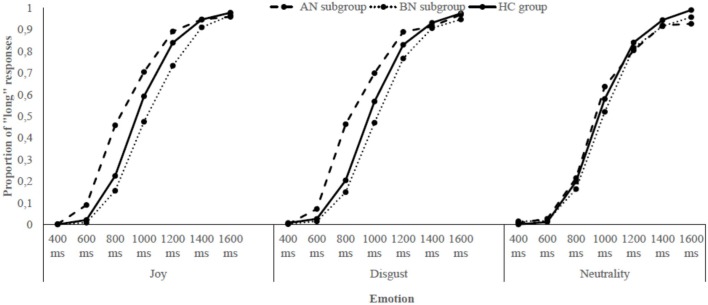
Proportion of “long” responses plotted against stimulus duration for each emotional condition and each subgroup of participants.

The data of the two bisection tasks – the one with joyful and disgusting food pictures and the one with a neutral object picture – were combined to compare the influence of these emotions on time perception. First, for each participant of both groups, the proportion of “long” responses (p[long]) for each stimulus duration and for each emotional condition was calculated. Then, the p[long] was plotted against durations. An examination of the **Figure 2** revealed that the p[long] increased as a function of stimulus duration for both groups, indicating that participants seemed to estimate time adequately (i.e., the longer the stimulus duration was, the more likely women responded “long”). However, the psychophysical functions were not distinctly shifted toward the left or the right, proposing no clear time distortion due to emotions. To explore the specificities of the ED diagnoses, the same procedure was applied within each ED subgroup: the p[long] was calculated and plotted against durations for women with AN and BN, in comparison with data of the HC group. A look at the **Figure 3** indicated, in addition to an effect of Duration, that there was a clear shift of the curve toward the left for the AN group when either joyful or disgusting food pictures were presented.

To further investigate these observations, two temporal indexes were calculated and analyzed: the bisection point (BP) and the Weber ratio (WR). The BP refers to the stimulus duration at which the participants would respond “short” or “long” with equal frequency (p[long] = 0.50). A higher BP means that durations are judged to be shorter, shifting the psychophysical function toward the right (i.e., underestimation of time). The WR reflects sensitivity to time. Lower WR indicates better performance (i.e., greater temporal sensitivity). Both measures were derived from a maximum likelihood fit of the proportion of “long” responses to the target durations on a cumulative gaussian curve. BP was equal to the mean parameter of the estimated gaussian curve, while the WR was equal to the standard deviation parameter of the estimated gaussian curve divided by the arithmetic mean of all target durations (i.e., 1000 ms). **Table 6** presents the BP and the WR values for each group, and for each ED subgroup.

**Table 6:** Bisection point (BP) and Weber ratio (WR) associated to emotional stimuli for groups of eating disorders (ED) and healthy controls (HC), and for anorexia (AN) and bulimia nervosa (BN) subgroups of ED.

Stimulus	ED group	HC group	AN subgroup	BN subgroup
**BP**
Joyful food	986.30 (162.91)	983.81 (117.99)	896.31 (167.53)	1055.52 (125.17)
Disgusting food	988.95 (165.92)	995.53 (134.56)	904.78 (182.15)	1053.69 (123.05)
Neutral objects	1014.31 (136.07)	990.75 (117.52)	1002.93 (186.53)	1023.06 (87.50)
**WR**
Joyful food	0.22 (0.07)	0.20 (0.06)	0.21 (0.09)	0.22 (0.06)
Disgusting food	0.22 (0.08)	0.21 (0.05)	0.21 (0.11)	0.23 (0.04)
Neutral objects	0.23 (0.09)	0.18 (0.05)	0.22 (0.10)	0.24 (0.09)


**Values represent means (standard deviations).**

A mixed-design analysis of variance (ANOVA) was conducted on the BP, with Emotion (J, D, and N) as a within-subjects factor and Group (ED and HC) as a between-subjects factor. For this analysis (and all the subsequent), the alpha level was fixed at 0.05 and corrections (Greenhouse–Geisser, Bonferroni) were applied when needed. The ANOVA revealed neither significant main effects of Emotion (*p* = 0.370) and Group (*p* = 0.867), nor significant Emotion × Group interaction (*p* = 0.436; see **Table 7** for the complete results). To investigate the presence of possible differences on the BP at the ED subgroups level, another ANOVA was carried out, comparing data of women with AN, BN, and without ED. The analysis showed no significant main effects of Emotion (*p* = 0.118) and Subgroup (*p* = 0.129), but the Emotion × Subgroup interaction was significant (*p* = 0.004; see **Table 7**). The decomposition of this interaction indicated that for joyful food pictures, women suffering from AN showed a lower BP than participants with BN (*p* = 0.019). A trend was noted for disgusting food images too (*p* = 0.052). However, there was no significant difference between AN and BN for the neutral object (*p* = 1.00). In other words, compared to women with BN, participants with AN significantly overestimated the duration of joyful food, and tended to overestimate the duration of disgusting food. Moreover, *post hoc* tests revealed that for the AN subgroup, the BP associated to joyful and disgusting food pictures were lower than the one linked to the neutral object picture (*p* = 0.005, *p* = 0.005). Thus, in comparison to the neutral stimulus, people suffering from AN overestimated the duration of food.

**Table 7:** Results of the ANOVAs for the temporal bisection task and the duration discrimination task.

	ED vs. HC			AN vs. BN vs. HC		
		
Effect	*df*_1_, *df*_2_	*F*	η^2^	*df*_1_, *df*_2_	*F*	η^2^
**Temporal bisection task**	**BP**					
Emotion	1.17, 51.66	0.88	0.02	1.22, 52.39	2.45	0.05
Group	1, 44	0.03	<0.01	2, 43	2.15	0.09
Emotion × Group	1.17, 51.66	0.68	0.02	2.44, 52.39	5.47^∗^	0.20
	**WR**					
Emotion	1.71, 75.06	0.28	0.01	1.69, 72.67	0.14	<0.01
Group	1, 44	2.30	0.05	2, 43	1.35	0.06
Emotion × Group	1.71, 75.06	2.78^†^	0.06	3.38, 72.67	1.58	0.07
**Duration discrimination task**	**Proportion of “long” responses**					

Emotion 1	1.75, 76.97	0.98	0.02	1.73, 74.35	1.19	0.03
Emotion 2	2, 88	0.19	<0.01	2, 86	0.22	<0.01
Group	1, 44	0.37	0.01	2, 43	0.32	0.02
Emotion 1 × Group	1.75, 76.97	1.58	0.04	3.46, 74.35	1.09	0.05
Emotion 2 × Group	2, 88	0.16	<0.01	4, 86	0.89	0.04
Emotion 1 × Emotion 2	2.90, 127.65	1.25	0.03	2.91, 125.05	1.35	0.03
Emotion 1 × Emotion 2 × Group	2.90, 127.65	0.74	0.02	5.82, 125.05	0.66	0.03
	**Proportion of correct responses**					
Emotion 1	2, 88	2.34	0.05	2, 86	2.41	0.05
Emotion 2	1.57, 69.18	2.30	0.05	1.61, 69.11	2.22	0.05
Group	1, 44	4.74^∗^	0.10	2, 43	2.33	0.10
Emotion 1 × Group	2, 88	0.52	0.01	4, 86	0.28	0.01
Emotion 2 × Group	1.57, 69.18	0.58	0.01	3.22, 69.11	1.61	0.07
Emotion 1 × Emotion 2	4, 176	1.43	0.03	4, 172	1.79	0.04
Emotion 1 × Emotion 2 × Group	4, 176	1.30	0.03	8, 172	0.97	0.04


*ED, eating disorders group; HC, healthy controls group; AN, anorexia nervosa subgroup; BN, bulimia nervosa subgroup; BP, bisection point; WR, Weber ratio. ^∗^Significant effect (alpha level adjusted with a Bonferroni correction when needed). ^†^Marginally significant effect (alpha level adjusted with a Bonferroni correction when needed).*

A mixed-design ANOVA was conducted on the WR, with Emotion (J, D, and N) as a within-subjects factor and Group (ED and HC) as a between-subjects factor. The analysis showed no significant main effects of Emotion (*p* = 0.721) and Group (*p* = 0.136), but a marginally significant Emotion × Group interaction (*p* = 0.076; see **Table 7**). *Post hoc* analyses indicated that, for the neutral object, the WR of the ED group was higher than the one of the HC group (*p* = 0.026), which means that women suffering from an ED had more difficulty to discriminate time (lower level of temporal sensitivity). Finally, to test the WR across the ED subgroups, a second ANOVA was run. However, neither the main effects of Emotion (*p* = 0.839) and Subgroup (*p* = 0.271), nor the Emotion × Subgroup interaction (*p* = 0.196), was significant or marginally significant (see **Table 7**).

#### Duration Discrimination Task

The data of the discrimination task were analyzed in terms of perceived duration and discrimination level (or sensitivity). The perceived duration is the probability of responding “long” (p[long]), which indicates, for each experimental condition (i.e., each pair of stimuli), whether the second picture presented is judged as shorter or as longer than the first one. The discrimination level is the probability of responding correctly (p[correct]), that is responding “long” when the duration of the second picture is effectively longer that the first one. Higher proportion of correct responses means greater temporal sensitivity. **Table 8** presents, for each pair of images, the p[long] and the p[correct] for the ED and HC groups, and for the ED subgroups.

**Table 8:** Proportion of “long” and correct responses associated to each pair of images, for groups of eating disorders (ED) and healthy controls (HC), and for anorexia (AN) and bulimia nervosa (BN) subgroups of ED.

Stimuli	ED group	HC group	AN subgroup	BN subgroup
**Proportion of “long” responses**				
Joy–Joy	0.52 (0.14)	0.52 (0.07)	0.52 (0.14)	0.52 (0.15)
Joy–Disgust	0.48 (0.16)	0.50 (0.11)	0.47 (0.14)	0.49 (0.18)
Joy–Neutrality	0.52 (0.17)	0.50 (0.10)	0.52 (0.16)	0.51 (0.19)
Disgust–Joy	0.52 (0.16)	0.48 (0.10)	0.50 (0.12)	0.53 (0.20)
Disgust–Disgust	0.52 (0.14)	0.48 (0.07)	0.49 (0.13)	0.55 (0.14)
Disgust–Neutrality	0.50 (0.18)	0.49 (0.06)	0.48 (0.16)	0.52 (0.20)
Neutrality–Joy	0.53 (0.18)	0.49 (0.11)	0.50 (0.16)	0.54 (0.20)
Neutrality–Disgust	0.55 (0.18)	0.50 (0.12)	0.51 (0.18)	0.58 (0.18)
Neutrality–Neutrality	0.51 (0.13)	0.50 (0.13)	0.53 (0.13)	0.50 (0.13)
**Proportion of correct responses**				
Joy–Joy	0.79 (0.11)	0.82 (0.09)	0.79 (0.13)	0.79 (0.10)
Joy–Disgust	0.77 (0.11)	0.82 (0.09)	0.75 (0.11)	0.78 (0.12)
Joy–Neutrality	0.79 (0.13)	0.84 (0.09)	0.80 (0.13)	0.78 (0.13)
Disgust–Joy	0.76 (0.13)	0.82 (0.08)	0.76 (0.12)	0.75 (0.14)
Disgust–Disgust	0.78 (0.10)	0.81 (0.10)	0.74 (0.08)	0.80 (0.11)
Disgust–Neutrality	0.74 (0.11)	0.83 (0.10)	0.76 (0.08)	0.73 (0.12)
Neutrality–Joy	0.75 (0.11)	0.79 (0.10)	0.76 (0.10)	0.74 (0.12)
Neutrality–Disgust	0.74 (0.13)	0.81 (0.12)	0.73 (0.11)	0.74 (0.15)
Neutrality–Neutrality	0.79 (0.10)	0.83 (0.09)	0.78 (0.10)	0.80 (0.11)


**Values represent means (standard deviations).**

A mixed-design ANOVA was performed on the p[long] with Emotion of the first picture (i.e., Emotion 1: J, D, and N) and Emotion of the second picture (i.e., Emotion 2: J, D, and N) as repeated variables, and Group (ED and HC) as a non-repeated factor. For this analysis (and all the subsequent), the alpha level was fixed at 0.05 and corrections (Greenhouse–Geisser, Bonferroni) were applied when needed. The ANOVA did not reveal any effect: neither main effects (Emotion 1, *p* = 0.371; Emotion 2, *p* = 0.825; Group, *p* = 0.547), nor interaction effects (Emotion 1 × Group, *p* = 0.215; Emotion 2 × Group, *p* = 0.853; Emotion 1 × Emotion 2, *p* = 0.295; Emotion 1 × Emotion 2 × Group, *p* = 0.523) were significant (see **Table 7**). Next, to test the presence of differences at the ED subgroups level, another ANOVA was carried out. Once more, no main and interaction effects were exposed (Emotion 1, *p* = 0.305; Emotion 2, *p* = 0.803; Subgroup, *p* = 0.727; Emotion 1 × Subgroup, *p* = 0.365; Emotion 2 × Subgroup, *p* = 0.473; Emotion 1 × Emotion 2, *p* = 0.261; Emotion 1 × Emotion 2 × Subgroup, *p* = 0.678; see **Table 7**).

For sensitivity, similar ANOVA designs were used. The first ANOVA showed no effect of Emotion 1 (*p* = 0.102) and Emotion 2 (*p* = 0.119), but the Group effect was significant (*p* = 0.035; see **Table 7**). More precisely, women suffering from an ED had a lower probability of responding correctly than the HC group (*p* = 0.035). However, none of the interaction effects was significant (Emotion 1 × Group, *p* = 0.598; Emotion 2 × Group, *p* = 0.525; Emotion 1 × Emotion 2, *p* = 0.225; Emotion 1 × Emotion 2 × Group, *p* = 0.273). The second ANOVA conducted with the subgroups showed no main and interaction significant effects (Emotion 1, *p* = 0.096; Emotion 2, *p* = 0.126; Subgroup, *p* = 0.110; Emotion 1 × Subgroup, *p* = 0.894; Emotion 2 × Subgroup, *p* = 0.191; Emotion 1 × Emotion 2, *p* = 0.132; Emotion 1 × Emotion 2 × Subgroup, *p* = 0.461; see **Table 7**).

### Influence of Non-temporal Factors

Correlation analyses on the entire sample were accomplished to verify and estimate the relation between the participants’ performance on temporal tasks, their clinical characteristics (i.e., BMI, ED features, actual mood, depressive and anxiety symptoms), their level of hungriness and their cognitive abilities. The performance on temporal tasks was explored according to the four indexes mentioned earlier, namely the BP, WR, p[long] and p[correct], with all emotions pooled together (i.e., joy, disgust, and neutrality). Because some variables were not normally distributed, Spearman correlations were executed. The alpha level was set at 0.05.

For the bisection task, there was no significant or marginally significant link between the BP and participants’ clinical characteristics, appetite and performance on neuropsychological tests. However, the WR significantly correlated with: BMI (*r*_s_ = 0.30, *p* = 0.046), Spatial Span Forward (*r*_s_ = 0.37, *p* = 0.013), Digit Span Backward (*r*_s_ = 0.33, *p* = 0.025), CWI Part 2 (*r*_s_ = -0.36, *p* = 0.014), CPT-II Hit RT SD (*r*_s_ = 0.33, *p* = 0.026) and CPT-II RT ISI Change (*r*_s_ = 0.45, *p* = 0.002). For the discrimination task, the p[long] was significantly associated with CPT-II Hit RT SD (*r*_s_ = 0.29, *p* = 0.047), and showed a marginally significant association with CPT-II Hit RT (*r*_s_ = 0.29, *p* = 0.053). The p[correct] significantly correlated with: TMT Part 1 (*r*_s_ = 0.45, *p* = 0.002), CPT-II Omissions (*r*_s_ = -0.29, *p* = 0.049), CPT-II Hit RT SD (*r*_s_ = -0.35, *p* = 0.019), CPT-II Hit RT ISI Change (*r*_s_ = -0.46, *p* = 0.001). It showed also a marginally significant relation with: RRS (*r*_s_ = -0.29, *p* = 0.050), EDE-Q (*r*_s_ = -0.29, *p* = 0.052), Digit Span Sequencing (*r*_s_ = -0.29, *p* = 0.053), CWI Part 2 (*r*_s_ = 0.28, *p* = 0.061) and CPT-II Hit RT (*r*_s_ = -0.26, *p* = 0.082).

To push further the reasoning about the influence of non-temporal factors on participants’ temporal performance, ANCOVAs were run^2^. In other words, we were interested to see if the statistical control of these factors would impact the differences between groups and subgroups of women. If yes, we could then infer that the observed differences seemed to be due to these factors. For each of the ANCOVAs performed, the covariates were the measures significantly correlated with the temporal index investigated, as presented above (i.e., the marginally significant relations were not included). Because no measure was correlated with the BP, no ANCOVA was performed for this index. The alpha level was fixed at 0.05 and corrections (Greenhouse–Geisser, Bonferroni) were applied when needed.

For the bisection task, the control of the influence of BMI and specific cognitive abilities (i.e., alertness constancy, vigilance, processing speed, and working memory) on the WR made the marginally significant Emotion × Group interaction effect disappeared (*p* = 0.210). More precisely, for the neutral condition (i.e., pictures of an object), the ED group, in comparison to the HC group, showed no more difficulty to discriminate time (*p* = 0.572). However, at the subgroups level, the inclusion of covariates did not change the results: the ANOVA and the ANCOVA showed no significant or marginally significant effect.

For the discrimination task, there was no consequence, on the results of the analyses, of removing the influence of alertness (constancy) on the p[long]: both the ANOVA and the ANCOVA, performed for groups and subgroups of participants, showed no significant or marginally significant effect. However, for the p[correct], the Group effect previously found with the ANOVA was lost after controlling for the participant’s cognitive abilities (i.e., selective attention, sustained attention, alertness constancy, vigilance; *p* = 0.485). In this sense, the ANCOVA showed that time sensitivity of ED and HC participants was no longer different. However, at the subgroups level, the results remained unchanged (once again, no effect was found).

## Discussion

The main objective of the present experiment was to study the emotional reactions of women suffering from an ED. In comparison of using traditional self-reported measures, which are prone to some biases, we worked with a different method, namely the presentation of food pictures and judgements about the duration of these presentations. The time perception perspective adopted is based on the fact that timing is sensitive to emotions and that temporal distortions give insights about how the environment is processed. Temporal bisection and duration discrimination tasks were used, which involved pre-rated joyful and disgusting food pictures and neutral object pictures.

The results demonstrated that, for both tasks, women with an ED, when pooled together no matter their diagnostic, did not show any time distortion when food pictures were presented, compared to object pictures and to HC. However, in the bisection task, when the ED group was split in function of diagnostic, women suffering from AN overestimated the duration of joyful and disgusting food pictures in comparison to neutral ones. Also, they perceived durations of joyful food pictures as longer than did women with BN, and tended to judge durations of disgusting pictures as longer too. HC, for their part, did not show any time distortion, for both tasks, in the food conditions compared to the object condition. In addition, the results showed that women suffering from an ED presented a lower temporal sensitivity than HC. In the bisection task, that was limited to neutral pictures, but in the discrimination task, this lower sensitivity occurred with both food and neutral pictures.

The lengthened-duration effect observed in women with AN for food pictures, no matter their initial valence, suggests a general reaction of fear to their presentation. According to the view of Angrilli et al. (1997) about the interaction between valence and arousal on time perception, an overestimation of durations is caused by a rise of the arousal level in response to a negative cue. More precisely, a threatening stimulus generates an elevation of the arousal level by an automatic response of the amygdala, which in turn speeds up the rhythm of the internal clock and activates the defensive system (Lang et al., 1997; LeDoux, 2000). More intense is the emotion of fear elicited by a threatening cue, more elevated is the arousal response to it (Lang et al., 1998; Bradley et al., 2001). In that regard, the overestimation of the presentation duration of food pictures by women with AN appears to be due to a rise of their arousal level, consecutive to a fear reaction. In other words, for these women, viewing food pictures seems to be strongly unpleasant, and even anxiety-provoking. That result is supported by behavioral experiments, which have shown that women suffering from an ED, particularly from AN, rate food pictures as less pleasurable and more fearful than HC (Rodríguez et al., 2007; Giel et al., 2011; Hay and Katsikitis, 2014). It is also supported by many psychophysiological studies that have demonstrated that exposure of AN women to food stimuli elicits an increase in their arousal level, as measured by heart rate, skin conductance and eye blink startle response (e.g., Perpiña et al., 1998; Gordon et al., 2001; Gorini et al., 2010), suggesting an automatic and unconscious reaction of anxiety. Similarly, fMRI researches have revealed that presentation of food pictures to women with AN increases their amygdala, medial prefrontal cortex (including the anterior cingulate) and insula activations (Ellison et al., 1998; Uher et al., 2003, 2004), three cerebral areas related to innate signals of fear (for review, see Damasio, 1994; Adolphs, 2013; Silva et al., 2016). Finally, the time distortions caused by a fear reaction in participants with AN is not without reminding those of spider-fearful people for which the durations of phobic stimuli are overestimated (Watts and Sharrock, 1984; Buetti and Lleras, 2012). For example, Tipples (2015) showed that the presentation of threatening stimuli to individuals especially reactive to them, as pictures of spiders for high phobic individuals, generates a rapid reaction of fear and a prompt rise of the arousal level, thus speeding up the internal clock, which results in increasing biases toward “long” responses. In brief, as exposed by studies with different methodologies, food pictures appear to be synonyms of fright for women suffering from AN.

### Outstanding Questions About Temporal Distortions Demonstrated in the Bisection Task

Two questions emanate from the lengthened-duration effect observed in AN in the bisection task. The first one is why BN participants did not show the same results, that is an overestimation of durations for food pictures in comparison to object pictures. A possible explanation is the fact that women suffering from BN are not as much frightened by food as people with AN are. According to the DSM-5 (American Psychiatric Association, 2013), AN is defined by an “intense fear of gaining weight or becoming fat,” a clinical criterion that does not characterize, *per se*, women with BN. To reduce their fear, women with AN rigidly avoid high-caloric food and are strongly reluctant to consume food outside a very narrow range (American Psychiatric Association, 2013; Savuskoski et al., 2016). Thus, many authors see evident overlaps between attitudes of patients with AN and those of anxious individuals in terms of worries/ruminations, intolerance of uncertainty, fear conditioning, avoidance strategies and reassurance seeking rituals (e.g., Pallister and Waller, 2008; Steinglass et al., 2011; Startup et al., 2013; Guarda et al., 2015; Kesby et al., 2017). Some of them even conceptualize AN as a phobia of food, weight gain and fat (Russell, 1967; Habermas, 1996; Crisp, 2006). By contrast, the relation of people suffering from BN with food stimuli does not seem so terrorizing. For instance, Léonard et al. (1998) demonstrated that, contrary to patients with AN, BN women did not show a rise of their arousal level (skin conductance) when confronted to a test-meal, suggesting an absence of fear reaction to it. Friederich et al. (2006), for their part, revealed that BN people did not only react without fear toward food pictures, but they also presented an exaggerated appetite response when exposed to them. Similarly, Drobes et al. (2001) showed that BN-like participants gave higher pleasantness ratings of food pictures than HC and AN-like participants. Considered all together, these studies propose that women suffering from BN do not feel threatened whilst viewing food pictures – rather they seem to resent a certain form of pleasure – and, thus, their arousal level does not increase abruptly in comparison to a base (non-food related) level. In that perspective, because a certain hedonic value of food seems preserved in BN women, it is also possible that these participants were more motivated than women with AN to execute the temporal tasks. In fact, Gable and Poole (2012) showed that a high-approach motivated state shortens the perception of time, causing time to be perceived as passing more quickly. So, if BN participants were more positively responsive to food pictures than AN women, they could have judge the durations of these stimuli as being shorter. Consequently, in the present study, the absence of an overestimation of durations of food pictures in comparison to those of objects (i.e., no rise of arousal) and the fact that BN women perceived the duration of food pictures shorter than AN participants did (i.e., positive reaction instead of fear) are better understood. In brief, for BN women, the absence of time distortions caused by food pictures in comparison to neutral ones, and the fact that their psychophysical functions for food are inversely shifted compared to those of AN, suggest that this ED subgroup reacts differently to that kind of stimuli. Therefore, when results of AN and BN subgroups are pooled together as a whole group for comparison to HC, the differences ED vs. HC can disappear.

The second and last question arising from the results is why the lengthened-duration effect observed with food pictures in the bisection task for women with AN did not also appear in the discrimination task. The different results are not so surprising considering that Gil and Droit-Volet (2011b) recently showed that time distortions due to emotions could depend on the task used. More precisely, they revealed that “the magnitude of the effect of emotions is reduced when more cognitive resources are required for the processing of time.” Thereby, a first way to explain the distinction between results in bisection and discrimination tasks is to explore mental operations required and cognitive processes solicited by each one. According to traditional models of bisection, participants who accomplish that task have to: (a) learn and store in long-term memory the S and L standard durations; (b) measure the length of the probe duration; (c) retrieve from memory the value of the S and L standard durations; (d) compare the probe duration to S and L standards; and (e) make a decision in function of the comparison done (Church and Deluty, 1977). Thus, from that classical perspective, the long-term memory processes of participants and their access seem very solicited. However, recently, some studies suggested that, instead of referring to S and L standard representations stored in memory, participants who perform a bisection task could rather conceptualize the probe durations as either S or L based on a criterion they form with the progression of the task, trial by trial (for review, see Kopec and Brody, 2010). In that view, the implication of long-memory processes could be diminished. At the opposite, the discrimination task is recognized to strongly rely upon working memory abilities (Mioni et al., 2013a). In that sense, participants must hold active the first duration and process, at the same time, the second duration presented (Grondin, 2008, 2010). When emotions of stimuli are manipulated (i.e., are not the same for the first and second pictures of a trial), it implies also to switch back and forth between the affective load associated with both durations when comparing them. So, with working memory capacities (maintenance and manipulation/updating), attentional resources (alertness, processing speed, selective, and sustained attention) and some executive functions (inhibition and cognitive flexibility) seem required when executing a discrimination task (Mioni et al., 2013a,b). In summary, the cognitive load related to the temporal discrimination task appears more important than the one required in the bisection task. In consequence, the influence of emotions on temporal processing could be reduced for that last task (Gil and Droit-Volet, 2011b), decreasing, by the same occasion, the opportunity to detect differences between conditions and groups. This hypothesis makes sense considering the fact that, in the present study, the discrimination task appeared to be significantly or marginally significantly correlated with more cognitive abilities than the bisection task, and the ED group, compared to the HC group, showed cognitive weaknesses in processing speed, attention, and executive functions. These weaknesses, which are in accordance with the literature (e.g., Steinglass and Glasofer, 2011; Jáuregui-Lobera, 2013; Weider et al., 2015), could have contributed, in the discrimination task, to decrease the influence of emotions provoked by food pictures on time perception (see section “Non-temporal Factors Influencing Performance on Temporal Tasks” for a more detailed explanation).

Besides the inherent processes of the temporal paradigms, another factor, the duration of the stimuli used, could contribute explaining the different results found for the bisection and the discrimination tasks. In fact, by analyzing affective reactions to emotional pictures, Codispoti et al. (2001) demonstrated that short presentations of unpleasant images resulted in less defensive activation (lower arousal) than sustained presentations of the same stimuli. Especially, the researchers showed that longer a subject is exposed to emotional pictures, particularly fearful images, stronger is his/her affective reaction (engagement) to them at a psychophysiological level. Because the discrimination task designed in the present study used short durations (400 and 482 ms) while the bisection task exploited a broader range of durations including longer presentations (400 to 1600 ms), the more sustained durations of pictures in the latter task could have favored the activation of the participants’ defensive system by promoting a deeper affective impregnation. Then, that impregnation, in turn, could have facilitated the production of time distortions.

### Different Methodological Choices: Different Results

A supplementary question emerging from the study is why the results for the HC group are not similar to those reported by Gil et al. (2009). More precisely, with a comparable bisection task (i.e., pleasant and disgusting food pictures, durations from 400 to 1600 ms), these authors showed an underestimation of food images, no matter their valence, compared to a neutral stimulus. They also demonstrated that the shortening effect was more marked for the disliked food pictures than for the liked ones. In our experiment, no time distortion was found for the HC group. These different results may emerge from some specific methodological variations in the studies.

Firstly, all food pictures used by Gil et al. (2009) led to low arousal. Consequently, according to Angrilli et al. (1997)’s point of view, time perception would then depend on controlled-attentional mechanisms. Viewed from a pacemaker-accumulator framework (internal clock), that means that the more the stimuli captured the participants’ attentional resources, the less the pulses were accumulated in the counter, resulting in shorter perceived durations. Thus, Gil et al. (2009) explained their shortening effect by the capacity of food pictures to detract participants’ attention from the passage of time. In the present study, all food pictures were high-arousing, so time perception did not depend mainly on the allocation of attention, but on the motivational-survival system: the more the stimuli represented a threat for participants, the more automatic and faster their durations were processed. As the survival of HC does not appear to be compromised by food pictures, they did not alter their ability to track the passage of time. Secondly, Gil et al. (2009) used a picture of a white oval as a neutral stimulus, whereas the present study used an object picture. The use of an image of a geometric figure, a stimulus different from a photograph, may have induced a bias in the processing of time information associated to it. In that sense, food pictures of Gil et al. (2009) appeared more visually complex than their neutral stimulus (e.g., 3D vs. 2D, plate with food on it vs. empty oval, multicolored item vs. one colored item) and, for that reason, they probably captured the participants’ attention. So, times distortions induced by the direction of subjects’ attention on food pictures could be due to the visual properties of these stimuli, not to their emotional nature *per se*. In our experiment, food pictures were more comparable to the neutral stimulus used (e.g., both were photos of 3D items, food and object images had similar sizes). As a result, attentional bias toward them – and time distortions deriving from them - was less probable. In the same vein, because HC participants in the study of Gil et al. (2009) were told to not eat 1 h before the testing phase, an extra attentional bias in favor of food pictures could have been induced by their non-sated state. In fact, hunger modulates attention to food-associated cues by producing an approach reaction (Loeber et al., 2013). Specially, attentional capture by food pictures is more marked for people who are not sated than for people who are (Lavy and van den Hout, 1993; Stockburger et al., 2008, 2009; Siep et al., 2009; Piech et al., 2010). Therefore, in Gil et al. (2009), the direction of participants’ attention toward food pictures and time distortions associated to them could have been generated by the degree of satiation, not by emotions provoked by food pictures. At the opposite, in the present research, participants were told to eat in the hour preceding the experiment and were tested at fixed moments, just after mealtimes, to reduce and control their hunger. Thus, it was less probable that they felt hungry (as indicated by the low cotes on the Likert-scales completed before the achievement of temporal tasks) and had an approach reaction toward food pictures due to their non-sated state.

Another methodological difference between the study of Gil et al. (2009) and the present one is the fact that women and men participated in the former one, while only women took part in the latter. Knowing that men show happier responses to food images than women (McNamara et al., 2008b), and considering that in low-arousing conditions, positive valence stimuli lead to an underestimation of time (Angrilli et al., 1997), the presence of men in Gil et al. (2009) could have contributed to the shortening effect observed. Inversely, because the present experiment was conducted with only women, a positive reaction toward food pictures was less plausible, especially considering that they were not hungry. Finally, Gil et al. (2009) did not document the affective state and the cognitive abilities of their HC participants, while it had been shown that these aspects could influence time perception (for review, see Droit-Volet, 2013; Teixeira et al., 2013; see also Tipples, 2008; Mioni et al., 2016b). As well, they did not evaluate if their participants presented ED-like concerns, attitudes and behaviors, which is an essential point to consider when working with food pictures and exploring emotions induced by them. Thus, it cannot be excluded that the performance on temporal tasks of Gil et al. (2009)’s participants was modulated by these factors.

### Non-temporal Factors Influencing Performance on Temporal Tasks

The second aim of the study was to identify and get a better understanding of the non-temporal factors that could contribute to explain the performance on the bisection and discrimination tasks. For that purpose, correlation analyses were run on the entire sample to estimate the relation between the women’s performance on temporal tasks, their clinical characteristics (i.e., BMI, ED features, actual mood, depressive, and anxiety symptoms), their level of hungriness and their cognitive abilities.

In the bisection task, the correlation analyses revealed that the bisection point (BP) was not associated with any of the variables tested. Similarly, the sensitivity to time (WR) was not significantly related to participant’s hungriness, actual mood and depressive or anxiety symptoms. However, it was correlated with the BMI. Concerning the cognitive functions involved in the task, it appears that the WR was moderately associated with spatial (maintenance component) and verbal (manipulation component) working memory, processing speed, stability of alertness and vigilance. In other words, participants’ time sensitivity improved with these cognitive processes. Besides, the present study showed that ED and HC groups were different on WR for the neutral stimulus, with women suffering from an ED having a higher WR (poorer sensitivity) than healthy women. The experiment also demonstrated that both groups were different on all cognitive variables significantly correlated with temporal sensitivity, except on working memory. Interestingly, when the measures related to the WR were statistically controlled for, the ED vs. HC difference disappeared. Thus, it cannot be excluded that, in the bisection task, the weaker temporal sensitivity of women with ED, in comparison to HC, was reduced (or explained) by their BMI and their cognitive weaknesses.

In addition, correlation analyses were performed for the discrimination task. The perceived duration of stimuli (p[long]) was related to only one measure: the stability of alertness capacity. The temporal sensitivity of participants (p[correct]) was positively associated with many cognitive processes, that is selective attention (visual scanning), sustained attention, alertness (constancy), and vigilance. A notable positive trend was also observed for verbal working memory (manipulation component), processing speed and reaction time (alertness level). Concerning the hungriness, the BMI and the clinical characteristics of women, only the restrained eating and the ED symptoms, as assessed by the RRS and the EDE-Q, were marginally and negatively related to time sensitivity. Once again, the fact that the ED and HC groups were different on most variables correlated with the p[correct] seemed to have contributed to explain why their performance on the task – when considering this index – was dissimilar. Indeed, controlling for measures significantly correlated to p[correct], namely their cognitive weaknesses, showed that ED and HC women were no longer different on their time sensitivity. Accordingly, the lower temporal discrimination level of participants suffering from an ED seemed to be due to the symptoms related to their diagnostic (i.e., restraint and concern) and, especially, to their cognitive weaknesses.

Considered all together, the results of the correlation analyses proposed that perceived duration (and time distortions), as reflected by the BP shift, is not associated with variations of BMI, ED restraint and concern, premorbid affective state, level of appetite and cognitive abilities *per se*. Something rather specific to the processing of temporal information at a psychophysiological level – like the running of the internal clock and its modulation by biological aspects (e.g., arousal level, defensive reaction) – seems to influence the perceived duration of stimuli and cause time distortions. The p[long] appears to be related to the stability of participants’ alertness, but this relation is likely not specific to perceived duration. It probably results from the higher cognitive load required by the discrimination task rather than from the alertness particularly. Inversely, the temporal sensitivity (i.e., WR, p[correct]) appears to be correlated with and impacted by non-temporal factors – or, said differently, by factors others than ones affecting the rhythm of the pacemaker – like ED symptoms and cognitive acuity. Consequently, the influence of these non-temporal factors on sensitivity to time is particularly important to consider, as it was demonstrated by two different tasks and it seconded the results of anterior studies showing the strong implication of attention, processing speed and working memory on time perception (for example, see Zélanti and Droit-Volet, 2011, 2012; Brown et al., 2013; Mioni et al., 2013a,b; Ogden et al., 2014; Droit-Volet et al., 2015). Furthermore, the ED vs. HC difference for the discrimination level disappeared when the cognitive weaknesses of women with ED were controlled for.

Finally, a detailed analysis of cognitive measures correlated to WR and p[correct], compared to those related to BP and p[long], may support the hypothesis of a heavier cognitive load required by the discrimination task than that required by the bisection one. In that sense, the correlation analyses showed that, in both tasks, the temporal sensitivity was associated with working memory, processing speed, constancy of alertness and vigilance. However, the discrimination task was also moderately-to-strongly linked to selective attention, sustained attention and reaction time (alertness level), three important cognitive processes that were not correlated (significantly or marginally significantly) with the bisection task. Therefore, by having a supplementary and heavier attentional component, the duration discrimination task seems to be more demanding than the bisection one, and so it could be more complex to perceive the influence of emotions on the processing of time when using it (Gil and Droit-Volet, 2011b). Such a more intense cognitive load could thus explain the difference in results between the discrimination and the bisection tasks on perceived duration, that is the detection of temporal distortions in the latter only. More precisely, as the bisection task seems to require less cognitive resources than the discrimination one, the effects of the emotions induced by food pictures were probably more easily detected. In other words, in that task, the participants were not “cognitively saturated”: some resources were still available for reacting to and processing the emotional value of the stimuli. In this line of thoughts, the absence of difference on the p[long] between the ED and HC groups in the discrimination task could come from the higher cognitive load associated to it, diminishing the possibility for the emotions to influence notably the perception of time, and then to cause temporal distortions. Moreover, if we consider that ED participants, in comparison to HC women, showed cognitive weaknesses in attention, processing speed, working memory, and executive functions, we can believe that they were even more “cognitively busy” with the discrimination procedure, and consequently, less likely to be affected by the emotions generated by food pictures. So, in that specific task, the detection of significant differences between food and object pictures for those participants was unlikely. Finally, because the ED subgroups were comparable on most of the cognitive measures used – AN women only tended to show a lower verbal working memory capacity that BN women (maintenance component) – neither was in a better “cognitive state” to allow more resources for the completion of the discrimination task. Along these lines, for both subgroups of ED, due to their similar cognitive profile, there was a limited window of opportunity for the emotion factor to influence the processing of temporal information. Consequently, no difference between emotional conditions and ED subgroups was observed.

### Limitations and Strengths of the Study

The generalization of the results of the present study may be reduced by the sample size and the fact that women of the ED group were not critically ill as revealed by their outpatient status and their BMI in the normal range. In fact, there was no difference on BMI between ED and HC participants. However, that is far from signifying that there were no notable symptoms of ED in the ED group. In that sense, the results on EDE-Q and RRS clearly demonstrated that the ED group presented significant symptoms of ED, and higher ED symptoms than the HC group. The absence of differences on BMI could be explained by three elements. The first one is, when AN and BN subgroups were pooled together in the ED group, the BMI of women with BN seemed to have inflated the mean BMI of the ED group. The second element is that HC participants were young university students, whereas BMI often increases with age (for example, see Williamson, 1993; Drøyvold et al., 2006; Reas et al., 2007). The third factor is the outpatient and community status of participants with an ED. In that sense, a very low BMI, which occurred frequently with serious medical complications, leads to an immediate medical support like hospitalization (Hay et al., 2014; Hilbert et al., 2017). Consequently, often, women suffering from an ED who receive outpatient services or who continue their daily activities like attending their university classes do not present a critical health status (i.e., their BMI is much closer to the normal range).

To the best of our knowledge, the present experiment is the first one investigating time perception of women suffering from an ED. This research is also very innovative for its adoption of a temporal perspective for studying the emotions elicited by food pictures in people with AN and BN. In fact, studying time distortions caused by the effects of emotions on time processing allowed us to better understand, in a subtle manner, how women with an ED react to food. In other words, this was done without relying on traditional self-reported procedures, which are regularly argued to be associated with important biases blurring conclusions (e.g., social desirability, low emotional awareness), and without relying on invasive psychophysiological techniques. In light of that, time perception could be considered as a new clinical tool for investigating, in an indirect but efficient way, the emotions of AN women toward food products. This way of approaching the problem was shown to be efficient, even with the patients who normally show much resistance when invited to share information about their illness or to verbalize their affective state. In addition, by using two different temporal tasks (bisection and discrimination), which lead to different results in term of time distortions (i.e., presence vs. absence), this experiment supports the growing literature about the fact that different temporal paradigms lead to different results (for example, see Gil and Droit-Volet, 2011b; Mioni et al., 2013a, 2014; Jisha and Thomas, 2015). It gives also extra thoughts about the complexities of time perception and specificities of methods used to examine it. Following this line of thoughts, in further researches, it could be interesting to explore the latent decision processes accompanying the responses of participants on temporal tasks. More precisely, some recent studies examine the response times of subjects, in complementarity of their choice proportions (e.g., p[long]), by using a drift-diffusion modeling (for relevant experiments, see Balci and Simen, 2014; Tipples, 2015; and for theoretical aspects, see Luzardo et al., 2013; Ratcliff, 2014; Vandierendonck, 2017). This perspective give access to additional material regarding the cognitive processing of temporal information and the dynamic influence of emotions on duration judgements, information that are detectable with difficulty if only choice proportions are analyzed. Such approach would require new data, however, as participants in the present study were not required to respond as quickly as possible. Finally, by adding affective and neuropsychological measures to temporal tasks, the present experiment contributes to a better understanding of how non-temporal factors influence (or not) perceived duration and temporal sensitivity.

## Conclusion

The present study showed that women suffering from AN presented an intense reaction of fear when they were confronted to food pictures, regardless of the emotion associated with said pictures (i.e., joy and disgust). In a temporal processing perspective, this emotional reaction was suggested by the overestimation of durations of food stimuli in comparison to object ones, due to a rise of arousal and the activation of the defensive system. The lengthened-duration effect observed in AN women was not detected in participants with BN, suggesting, for this ED subgroup, a less emotional negative reaction toward food, and even the preservation of a hedonic response to it. Thus, for women suffering from BN, food seems to have kept a form of appetent and motivational properties. Considered all together, these results support the idea of a distinctive functioning of the reward pathway in AN and BN (for example, see Davis and Woodside, 2002; Keating, 2010; Keating et al., 2012), and consolidate the thesis of etiological and symptoms similarities between AN and anxiety disorders (e.g., Pallister and Waller, 2008; Steinglass et al., 2011; Guarda et al., 2015; Kesby et al., 2017). Therefore, the elaboration of treatment strategies addressing these aspects seems essential to generate positive and long-lasting changes in women with an ED.

## Author Contributions

CG, SG, and CB designed the study, and CG ran it. CG and VL conducted the statistical data analyses. CG wrote the first draft of the manuscript. All authors contributed equally to the revision of the paper and then approved the final version of the manuscript.

## Conflict of Interest Statement

The authors declare that the research was conducted in the absence of any commercial or financial relationships that could be construed as a potential conflict of interest.

## Footnotes

1 Initially, the time perception tasks (bisection and discrimination) of this study were designed to be used with electroencephalographic (EEG) recordings. Consequently, an elevated number of trials per condition (for a better signal-to-noise ratio) and a long delay between the steps of the tasks (e.g., ITI, stimulus-answer delay; for viewing the apparition and the extinction of cerebral components) were planned.

2 In reference to the arguments of Miller and Chapman (2001) and those of Suckling (2010), we acknowledge that the use of ANCOVAs to “control for” or to “correct for” pre-existing group differences (e.g., cognitive abilities) when participants are not randomly assigned can be controversial. Accordingly, we think that a most appropriate way – in respect to the assumptions of the statistical tests and the conclusions they allow us to draw – to explore the influence of non-temporal factors on participants’ temporal performance could have been to run mediation analyses. However, as these analyses are based on structural equation modeling, the detection of significant effects requires a large sample size (low statistical power) and is strongly dependent of the variables studied/entered in the model. Because the present experiment had a small sample size and the selection of the measures to be investigated could have been very ambiguous considering their number and their interrelations, we decided not to perform these mediation analyses.
